# Differential Roles of Human 3R and 4R Tau in the Modulation of Inflammation and Development of Neuropathology

**DOI:** 10.1002/alz70855_105749

**Published:** 2025-12-23

**Authors:** Brian Spencer, Aaron Schueler, Daniel Sung, Robert A. Rissman

**Affiliations:** ^1^ Alzheimer's Therapeutic Research Institute, Keck School of Medicine, University of Southern California, San Diego, CA, USA; ^2^ Alzheimer's Therapeutic Research Institute, University of Southern California, San Diego, CA, USA

## Abstract

**Background:**

Alzheimer's disease (AD) is the most common tauopathy characterized by progressive accumulation of Aß and tau neuropathology. Tau is expressed in two major isoforms containing either 3 or 4 C‐terminal repeats, 3R and 4R. Despite tau isoforms occurring in roughly equimolar ratios in AD, the majority of research focus and developed mouse and *in vitro* models focus only on 4Rtau.

**Method:**

To generate a more complete model of AD tauopathy and understand specific tau isoform‐mediated neuropathology and neurodegeneration, we generated a transgenic mouse line expressing both 3Rtau and 4Rtau and determined how this impacted the timing and severity of neuropathological and behavioral changes.

**Result:**

3R/4Rtau bigenic mice expressed increased tau and phosphorylated tau in the hippocampus and cortex compared to single (3R or 4R) transgenic cohorts as early as 3‐months of age and this was accompanied with increased astrogliosis and microglial activation. 3R/4Rtau mice expressed phosphorylated tau similar to that found in AD patients including PTau181 and PTau 396/404. Bigenic mice had significantly greater behavioral deficits compared to either single transgenic littermates in spatial learning and memory as well as nest building, indicative of depression and/or cognitive deficits.

**Conclusion:**

This new mouse model of tauopathy more completely recapitulates the pattern, severity and accumulation of tau and associated neuropathology and behavioral changes observed in human tauopathies such as AD. 3R/4Rtau‐tg bigenic miceshould supplant existing single transgenic tau models for general validation of therapeutic targets and investigations of novel therapies on tauopathy endpoints.

